# Finite element analysis of the pelvis including gait muscle forces: an investigation into the effect of rami fractures on load transmission

**DOI:** 10.1186/s40634-018-0151-7

**Published:** 2018-09-03

**Authors:** Pierre-Louis Ricci, Stefan Maas, Jens Kelm, Torsten Gerich

**Affiliations:** 1 Research Unit in Engineering Sciences, Campus Kirchberg, Université du Luxembourg, 6 rue Richard Coudenhove-Kalergi, Luxembourg, L-1359 Luxembourg; 2 Chirurgisch-Orthopädisches Zentrum, Rathausstr 2, 66557 Illingen, Saar Germany; 3 Centre Hospitalier de Luxembourg, Service de Traumatologie, 4 rue Ernest Barblé, Luxembourg, L-1210 Luxembourg

**Keywords:** Pelvic ring fracture, Biomechanics of the pelvis, Physiological loadings of the gait, Muscle forces, Finite element analysis

## Abstract

**Background:**

The objective of the study is to investigate the load transmission within the pelvic ring under physiological loading during gait and to correlate these results with clinical findings. In a second approach, we analysed how load distribution is altered by fractures of the anterior pelvic ring.

**Methods:**

Muscle forces and joint reaction forces are calculated by inverse dynamics and implemented in a finite element pelvis model including the joints.

**Results:**

With the intact configuration and according to the moment of the gait, left and right superior and inferior rami show the highest stresses of the model, corresponding to the typical location of an anterior pelvic ring fracture. A superior ramus fracture induces larger stresses to the lower ramus and a slight increase of stresses on the posterior structures. A total disruption of anterior rami redirects the loads to the back of the pelvis and introduces significantly higher stresses on the posterior structures.

**Conclusions:**

This investigation enhances the understanding of the biomechanics of the pelvis and highlights the important role of the rami in load carrying and in maintaining integrity of the pelvic ring.

## Background

The osseous pelvis is a complex circular structure. The left and right ilium and the sacrum are linked at the level of the pubic symphysis anteriorly and by two sacroiliac joints posteriorly (Netter 2007). This pelvic ring, reinforced by muscles and ligaments (Schatzker & Tile 2005), enables load transfer from the lumbar spine to the lower extremities. These loads are higher in the dorsal aspect of the pelvis compared to the anterior part. Hence, the anterior structures are more filigrane and prone to fracture.

Clinically, we are mainly confronted with osteoporotic insufficiency fractures of the anterior or/and posterior pelvic ring in a geriatric population (Hill et al. 2001). For this entity, the term “Fragility Fractures of the Pelvis” (FFP) has been established. The incidence of these fractures of the pelvis increased by 460% between 1970 and 1997; for the period between 2005 and 2025, it is estimated to increase by 56% (Burge et al. 2007, Kannus et al. 2000). This is not only a temporary debilitating situation but has an immediate impact on function, independency and survival rate. The 1-year mortality rate is estimated to be as high as 19% (Hill et al. 2001, Taillandier et al. 2003, Dodge & Brison 2010, Krappinger et al. 2010, Studer et al. 2013). Patients with a FFP above the age of 90 had a 1-year mortality of 39% (Krappinger et al. 2010). The overall 5-year mortality reached 54% and the authors observed a further increase with age and dementia (Kannus et al. 2000); after 10 years, the overall mortality rate reached 94%, which was statistically significantly higher than observed in an age-matched population (Van Dijk et al. 2010).

The high mortality rate goes along with a decrease of the functional status. One year after the fracture, 84% of the patients depended on walking aids and only 18% were able to live independently (Dodge & Brison 2010), 50% lost their previous autonomy (Breuil et al. 2008). In a continuously ageing population, this is not only of great interest among the scientific community but reflects an economic burden (Clement & Court-Brown 2014).

Therefore, it must be a continuous effort to investigate and understand the biomechanical parameters behind certain fractures types and the time-dependent development of pelvic disintegration and to develop strategies and techniques to stabilize FFP. In this context, numerous experimental studies have been performed during the last decades in order to compare different techniques (Berber et al. 2011, Prasan et al. 2012, Vigdorchik et al. 2012, Osterhoff et al. 2015). However, the complexity of the interaction between bone, ligament and muscle are poorly understood. Engineering tools such as Finite Elements (FE) offer a potential solution. Studies can now numerically focus on inaccessible locations and encourage further thinking to target clinical management (Savoldelli et al. 2009). Hence, many models with various problems rely on this numerical method (Liao et al. 2009, Kehe et al. 2013, Shi et al. 2014, Fan et al. 2015, Lei et al. 2015, Liu et al. 2015, Yao et al. 2015). However, it should be kept in mind that results depend on input parameters and that caution is therefore mandatory for interpretation (Viceconti et al. 2005) because these models are a simplification of physiological conditions (Hao et al. 2011).

To our knowledge, there is no literature investigating the biomechanics of the entire pelvis and the force transmission under physiological loadings of the gait induced by controlled muscle forces and hip joint reaction forces. Therefore, the objective of this study was the investigation of the force distribution in the pelvic ring during a normal gait movement. Hence, a non-fractured pelvis including joints was used to develop a FE model taking into account muscle forces and hip joint reaction forces obtained from an inverse rigid-body dynamics approach during normal walk. Moreover, models with either superior pubic ramus fracture or single sided anterior pelvic ring fracture were considered to further assess the stability of the pelvis and understand how anterior fractures alter the distribution of loadings.

## Methods

### Geometries

**Fig. 1:**
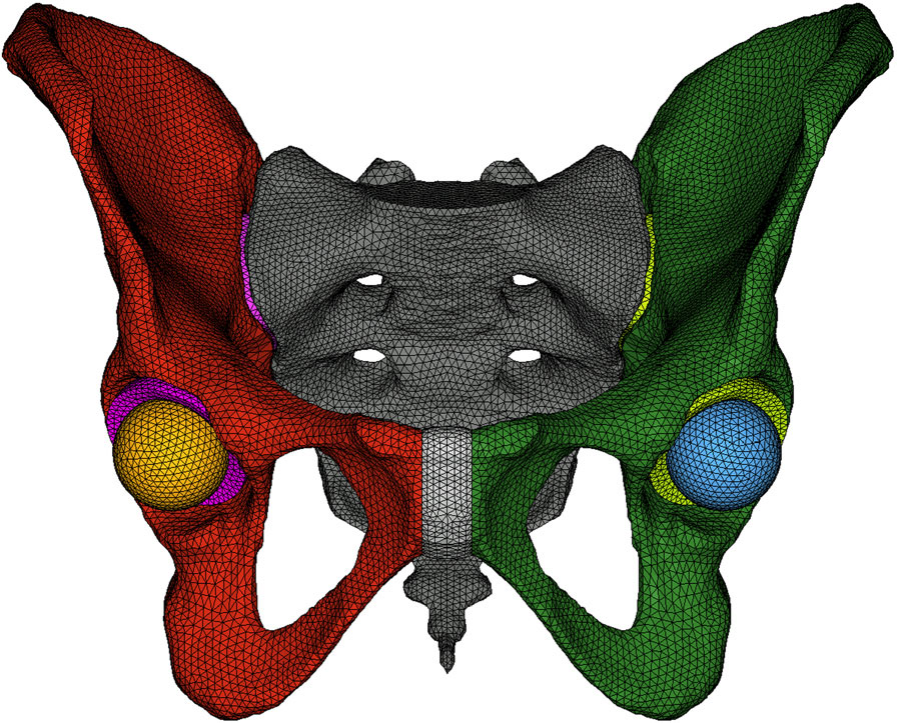
Geometries from the FE model of the non-fractured pelvis

DICOM images of an entire male adult type pelvis from OsiriX (Pixmeo, Geneva, Switzerland), including 360 CT-slices at 1,5 mm thickness taken with a Phillips Mx8000 CT scanner (KvP 140, X-Ray tube current 272, Exposure 227, Feet First Supine) were segmented using ITK-SNAP 3.2 software (University of Pennsylvania and University of Utah, USA). DICOM images were made available by OsiriX in their online image library exclusively for research and teaching. Consequently, those datasets are deprived from patient’s personal data (e.g.: age, height, body weight). Non-fractured external geometries of one hipbone and the sacrum were later imported into HyperMesh 12.0 (Altair Engineering, Troy, Michigan, USA) for both surface cleaning and mirroring to obtain a fully symmetrical intact pelvis considered suitable by experienced surgeons for conducting the study. Because of the difficulty in distinguishing soft tissues with Computed Tomography scans, both sacroiliac joints and the pubic symphysis were constructed in this very same software by linking the articular surfaces of bones according to anatomical observations from literature (Netter 2007, Becker et al. 2010). Two spheres were created to represent each femoral head. The free space between the femoral head and the acetabulum was considered as a new component, named “acetabular cap”, representing cartilage and other soft tissues in order to distribute joint reaction forces from the hip to the acetabulum. Geometries of hipbones, sacrum, PS, SI joints, acetabular caps and femoral heads used for FE analysis are shown on Fig. 1.

### Meshing, contact, mechanical properties

Obtained geometries were imported into ANSYS Workbench 16.2 (ANSYS Inc., Canonsburg, Pennsylvania, USA) for FE analysis. Since the study mainly focused on bones and joints, the complex modelling of the ligaments was ignored. Therefore, all the contacts between components were considered as fully bounded in order to keep the pelvis assembled. Patch independent algorithm together with quadratic tetrahedral elements were used for meshing. Mechanical properties having a linear isotropic elastic behaviour (Young’s modulus E and Poisson’s ratio ν) were defined as given in Table 1. Due to the investigation of the global load distribution within the pelvis rather than threshold values of stresses, no clear distinction was made between cortical and cancellous bones for simplification purposes. Nevertheless, an averaged Young’s Modulus E was used to take into account those two types of bones (Ravera et al. 2014).

**Table 1:** Size of elements and mechanical properties of components

	Component	Size of elements(mm)	Mechanical properties	
			E (MPa)	ν
Ravera et al. 2014	Left / Right Pelvis	2	7000	0,3
	Sacrum			
	Femoral head			
Fan et al. 2015	Pubic symphysis	1,5	5	0,495
Lei et al. 2015	Sacroiliac joints	1,5	350	0,495
Shi et al. 2014	Acetabular caps	2	12	0,42

### Boundary conditions

**Fig. 2:**
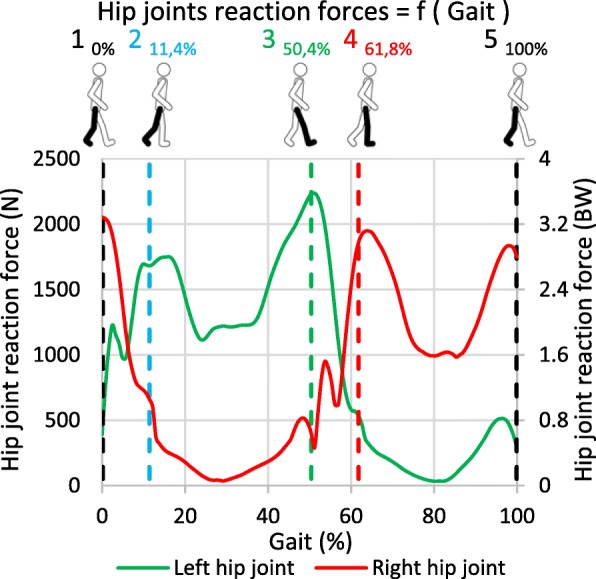
Hip joint reaction forces during gait

Physiological loading conditions were obtained by an inverse rigid-body dynamics analysis performed with AnyBody 6.0 (AnyBody Technology, Aalborg, Denmark). By using the available standard gait model already experimentally validated (Manders et al. 2007), all the forces applied to the pelvic ring originating from muscles, hip joints and the lumbosacral joint were calculated according to the gait of a healthy person (62 kg, 173 cm). Reaction forces at the pubic symphysis and at both sacroiliac joints were not calculated, as they are part of the pelvic ring. Figure 2 illustrates the resultant force from hip joints applied to the pelvis, in Newton (N) and BodyWeight (BW) scales, according to the percentage of gait cycle.

The study of the gait cycle was divided into several static key positions corresponding to peaks of reaction forces at the hip joints and represented by the vertical dashed lines with analogous numbers 1, 2, 3, 4 and 5. These precise instants of the gait, as illustrated by the walking subject, correspond to:

- Position 1 (0%): Left foot strike,
- Position 2 (11,4%): Right toe off,
- Position 3 (50,4%): Right foot strike,
- Position 4 (61,8%): Left foot off,
- Position 5 (100%): Left foot strike.

Position 1 and 2, maximal reaction forces peaks, represent important loading imbalances among left and right hips and are investigated in the current study. The analysis of position 3, 4 and 5 do not provide additional information compared to position 1 and 2: position 3 and 4 are the symmetries of position 1 and 2 whereas position 1 is the same as position 5.

Each force acting on the pelvis computed by inverse dynamics was decomposed into three components along space direction. These components were implemented into an ANSYS Workbench FE model as direct or remote forces applied on surfaces of the geometries of bones for muscles (according to anatomical muscles attachments (Netter 2007, Drake et al. 2014)) and to femoral heads as direct forces for left and right hip joint reaction forces. The global magnitudes are reported in Table 2 for information. For each position, pelvises in the FE model were oriented in space as in the inverse dynamics software.

**Table 2:** Applied forces (N) in position 1 and position 2

Joint / Muscle	Applied forces (*N*)			
	Pos. 1 (0%) Left foot strike		Pos. 2 (11,4%) Right toe off	
	Left	Right	Left	Right
Hip	391	1998	1663	645
Adductor	2	128	–	204
Biceps femoris	66	–	197	–
Erector spinae	28	44	133	140
Gemellus	8	40	32	16
Gluteus	63	512	848	–
Gracilis	17	8	–	26
Iliacus	43	169	–	116
Multifidi	18	15	36	23
Obliquus internus	112	85	24	66
Obturator	28	268	164	231
Pectineus	5	25	–	25
Piriformis	–	66	56	–
Psoas major	7	39	2	24
Quadratus femoris	2	25	2	58
Quadratus lumborum	14	11	4	30
Rectus abdominis	–		–	
Rectus femoris	–	494	34	241
Sartorius	47	127	–	80
Semimembranosus	137	–	78	–
Semitendinosus	127	–	38	–
Tensor fascia lata	–	88	13	39
Sum of forces (for information only)	1115	4142	3324	1964
	5257		5288	

A remote point to the lumbosacral articular surface was defined as the centre of the lumbosacral joint linking the model to the environment. No forces from the inverse dynamics analysis were applied to the location of this spherical joint. Nevertheless, reaction forces at this joint between both packages (AnyBody and ANSYS Workbench) were compared and used in the next section for assessment purposes of the developed model.

### Assessment of the model

It is difficult to compare different FE analyses when boundary conditions are highly varying. Validation among FE studies from literature is usually done based on experiments loading pelvises from cadavers (Dalstra et al. 1995, Anderson et al. 2005). Nevertheless, it can be questioned if current experimental setups manage to represent complex physiological configurations as the one presented in this paper and its 108 muscles boundary conditions to validate stresses and strains in the numerical model. Hence, it was decided to assess the reliability level of this simulated physiological configuration by comparing the reaction forces at the lumbosacral joint computed by inverse dynamics (AnyBody) with the reaction forces at the spherical joint calculated by FE analysis (ANSYS Workbench). This representation of equilibrium is summarized in Table 3 for both position 1 and 2. Variations between the FE analysis and the preliminary inverse dynamics study, considered as reference, appear acceptable and could be due to the different geometries between both software and the neglect of inertia forces of the gait during FEA. Hence, the obtained FE model is considered valid, confirming its ability in reproducing gait physiological loadings to the bones.

**Table 3:** Reaction forces at lumbosacral joint

Forces (N)	Lumbosacral joint			
	Pos. 1 (0%) Left foot strike		Pos. 2(11,4%) Right toe off	
	ID	FE	ID	FE
X	− 136	− 124	−103	− 155
Y	− 724	− 676	− 617	− 584
Z	−21	61	−52	23

Unlike the majority of the studies in literature where extremities of bones are fixed in translation, the present study uses a spherical joint to connect the model to the environment thereby allowing rotation along the three special directions. Hence, two sets of three 1 cm low stiffness springs (10 N/mm), oriented in the three directions of space, were inserted at both ischial tuberosities. Those six stabilisation springs were only used for numerical convergence reasons and do not influence the results by creating local stresses as their elongations are always below 1 cm per side; being insignificant when compared to the approximately 5,3 kN forces from Table 2 applied to the pelvis.

### Available models

One intact model and two models with rami fractures were used to investigate the biomechanics of the pelvis during the gait. Rami fractures were directly created on geometries under the supervision of an experienced surgeon. No contact was defined between both bone extremities at the location of the fracture. This led to the following configurations:

A. Non-fractured pelvis,
B. Right superior ramus fractured pelvis,
C. Right superior and inferior rami fractured pelvis.

**Fig. 3:**
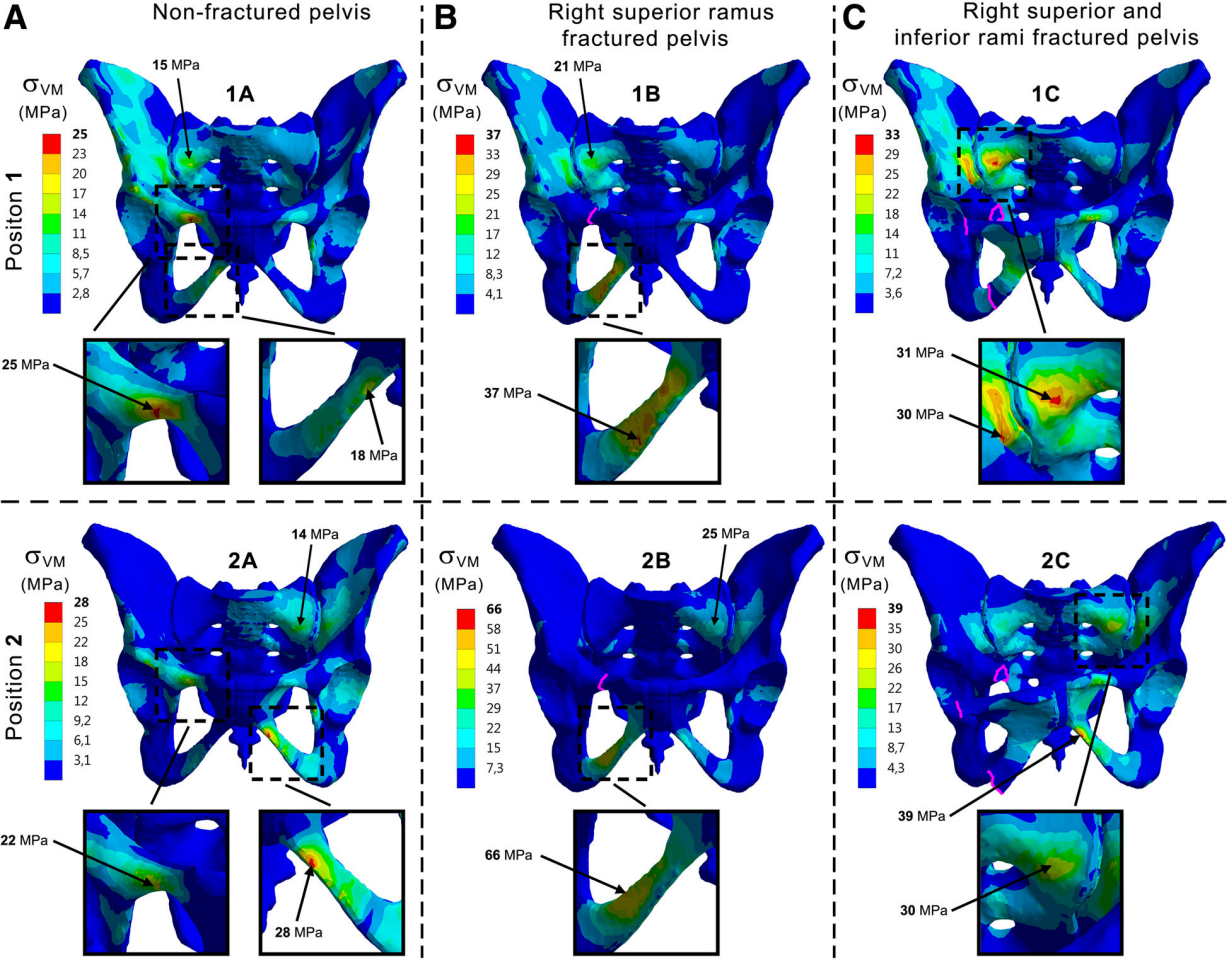
Frontal views of Von Mises stresses applied to the pelvis. **1**: Position 1. **2**: Position 2. **A**: Non-fractured pelvis. **B**: Right superior ramus fractured pelvis. **C**: Right superior and inferior rami fractured pelvis

**Fig. 4:**
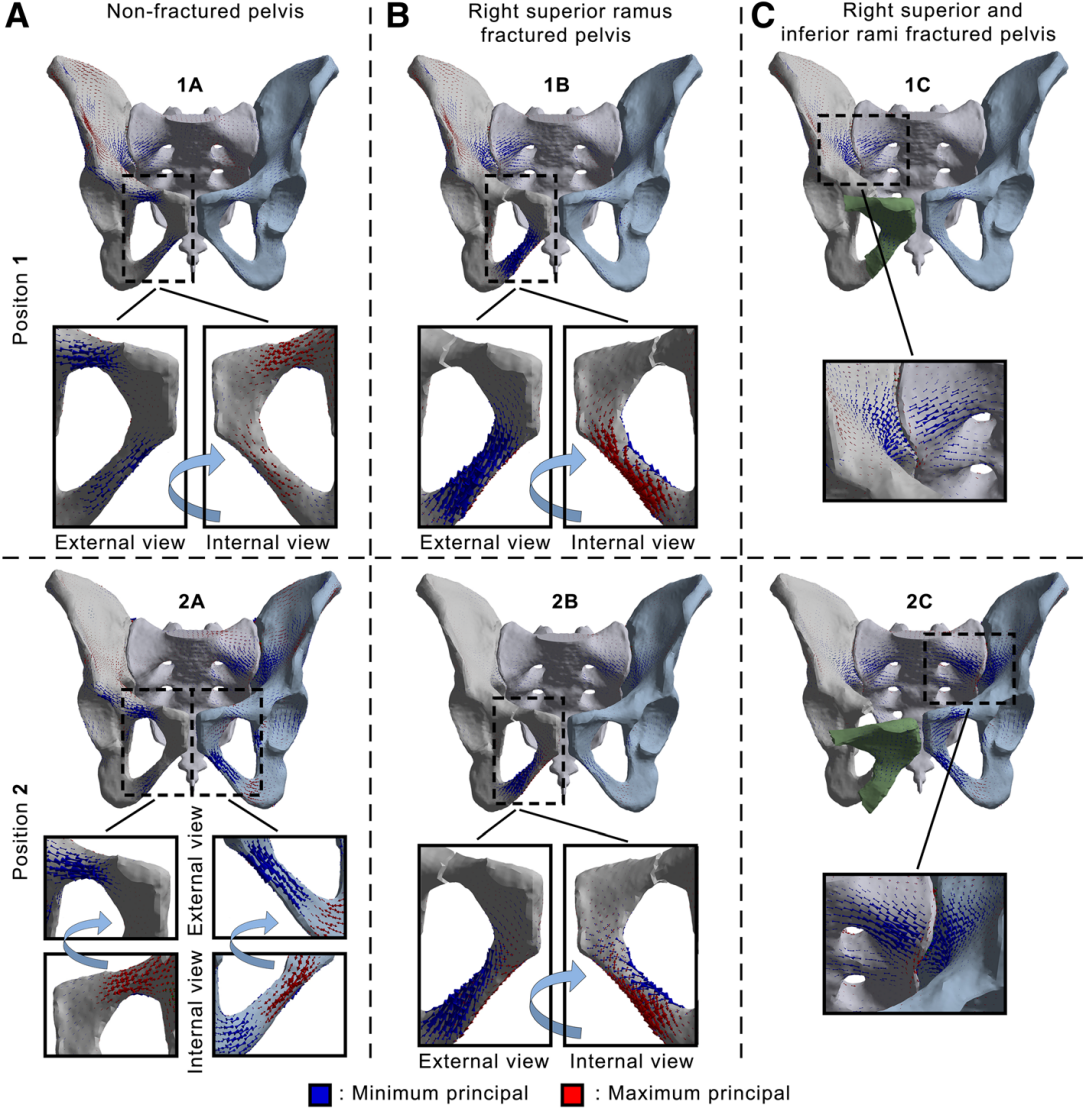
Frontal views of principal stresses vectors applied to bony structures. **1**: Position 1. **2**: Position 2. **A**: Non-fractured pelvis. **B**: Right superior ramus fractured pelvis. **C**: Right superior and inferior rami fractured pelvis

**Fig. 5:**
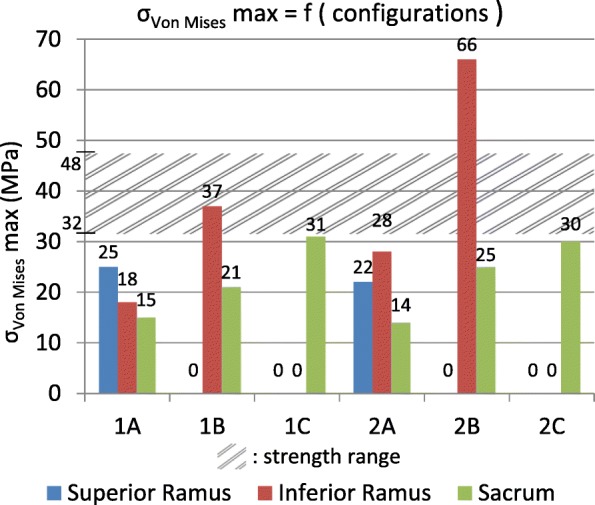
σ_Von Mises_ max in the models. **1**: Position 1. **2**: Position 2. **A**: Non-fractured pelvis. **B**: Right superior ramus fractured pelvis. **C**: Right superior and inferior rami fractured pelvis

Given that no fractured musculoskeletal model was available for inverse dynamics analysis, the three FE models have the same load distribution and fractures are therefore the only variation between models. Output criteria are Von Mises (VM) stresses on bony structures and joints, Fig. 3, to evaluate global distribution of stresses within the pelvis (with fractures lines represented in violet). In addition, principal stress vectors on bones, Fig. 4, were monitored to account for the way both hipbones and the sacrum are solicited. For ease of reading and comprehension, non-fractured pelvis (Model A) is first considered. Then, fractured configurations (Models B and C) are included to observe how load distribution is changing. Figure 5 summarizes the calculated existing stress values and is used for final assessment.

## Results

### Model a: Non-fractured pelvis

Globally, higher VM stresses were located on the right side of the pelvis in position 1 of the gait (left foot strike). The superior ramus experiences higher VM stresses (25 MPa) than the inferior ramus (18 MPa). Additionally, it may be noted the slight concentration of stresses with a maximum of 15 MPa on the right face of the sacrum and also on the right wing internally to the pelvic ring. Regarding principal stress vectors, the length is proportional to the absolute value, with red colour for tension and blue colour for compression. Therefore, it indicates a bending of rami by external compression and internal tension, with higher values on the right side. Compression zones are also internally located at the greater sciatic notch and sacrum.

When considering position 2 (right toe off) corresponding to a shift of load from right to left, the higher solicited rami are diagonally opposed: right superior and left inferior with close VM stresses values of respectively 22 and 28 MPa. The slight increase of stresses on the right face of the sacrum and the wing internally to the pelvic ring is now located to the left side and reaches 14 MPa. The principal stress vectors follow the same observation as for the VM stresses with diagonally opposed rami solicited. Bending is still present on those anterior branches, whereas compression to the internal structures of the back is located on the right side.

### Model B: Right superior ramus fractured pelvis

A noteworthy increase of VM stresses at the inferior ramus with significant values of 37 MPa is visible. A growth may also be noticed internally medial and lateral to the right sacroiliac joint with a 21 MPa peak. With this superior ramus fracture, the inferior ramus undergoes significant bending. The bony structures surrounding the right sacroiliac joint from the inside are continuously under compression.

In position 2, the increase of VM stresses is still present on the inferior ramus but with even higher values than previously, reaching now 66 MPa. The internal region with higher VM stresses internally on the back of the ring is now located on the left side with a value of 25 MPa. As in position 1 where the region of the right sacroiliac joint experiences compression, the same observation can be made here in position 2 but with the left side rather than the right one involved.

### Model C: Right superior and inferior rami fractured pelvis

With a total fracture of both right rami in position 1, a growth of VM stresses until 31 MPa is induced internally on the back of the pelvis structures, especially on the right sacrum, as it also may be seen with the principal stress vectors illustrating compression. Because of fracture dividing the right pelvis in two parts, the left pubic bone is pulled by the surrounding muscles. This total rupture creates an opening of the front pelvis by significantly deforming the pubic symphysis and bringing concentrations of VM stresses to the left rami.

Same observations can be made for the position 2 concerning the pubic symphysis and the left rami. Nevertheless, the internal region with higher VM stresses at any side of the right sacroiliac joint is shifted to the left of the pelvis and reaches 30 MPa. The main compressive principal stresses are also changing side to reach the internal surrounding region of the left sacroiliac joint.

## Discussion

The number of FE studies dealing with pelvis is constantly growing over the past decades (Liao et al. 2009, Kehe et al. 2013, Shi et al. 2014, Fan et al. 2015, Lei et al. 2015, Liu et al. 2015, Yao et al. 2015) as it allows more flexibility than experimental setups. Such simulations include various simplifications adverse to physiological configurations (e.g: fixed extremities, no inclusion of muscular/joints reaction forces) and parallelism with reality should be done carefully when it comes to clinical relevance. The boundary conditions of the model should be related to physiological loadings as faithfully as possible so that the stress distribution corresponds the real physical situation and following Saint-Venant’s principle.

Many authors considered in their simulations a fixed extremity, e.g. the first sacral body (Yao et al. 2015), the proximal femur (Fan et al. 2015, Lei et al. 2015, Shi et al. 2014, Liu et al. 2015), or the acetabulum (Liao et al. 2009, Kehe et al. 2013). With this, those studies intended to simulate by single and double loadings the double leg stance (Fan et al. 2015, Lei et al. 2015, Shi et al. 2014, Liu et al. 2015) or the weight of the torso (Kehe et al. 2013). However, it could be questioned if those configurations do not get closer to in vitro experiments rather than in vivo environments because of simplified boundary conditions altering the loads distribution and creating concentration of stresses close to fixations. Phillips et al. (2009) reproduced the single leg stance simulations of Anderson et al. (2005) and Dalstra et al. (1995) and highlighted non-negligible differences with a model they developed simulating muscles and ligaments by the use of springs instead of simple loadings and fixations. Authors introduced the loadings at the femoral heads and forced therefore the springs to exert compression forces as reactions to simulate the muscles. In the present paper, it was decided not to include muscle forces as reaction forces but rather as active forces to consider physiological loadings when walking. Realistic attachments of muscles were taken into account when applying muscle forces extracted from the inverse dynamics study of the gait cycle. The full body gait model from AnyBody used in this study was validated by Manders et al. (2007) by comparison with studies of Bergmann et al. (2001) and Brand et al. (1994), which implemented instrumented hip implants in patients. The close correlation between hip joint reaction forces from experimental data and from numerical calculation comforted the choice of including muscular forces as controlled forces and therefore considering a more complex loading of the gait for an improved relevance of the model. Nevertheless, it was chosen to avoid the modelling of the ligaments because of this paper focusing on load transmission across the bony structures of the pelvis. Hence, bounded contacts between components to keep the integrity of the pubic symphysis and both sacroiliac joints were used. Moreover, it is believed that distinction between the cortical and spongeous bones would not have a compulsory role on the distribution of stresses, but rather on the magnitude of peak values. Consequently, the boundary between those two types of bones was not defined and consequently the bone was considered as a single material with averaged mechanical properties (Ravera et al., 2014).

The present study aims at improving the common understanding of the behaviour of the pelvis during walking, and more globally the biomechanics of the pelvic ring in a healthy configuration and with anterior fractures. The stress concentrations at the superior and inferior rami indicate that those branches allow for load transmission between the left and right side in a healthy pelvis (case A in Fig. 5). The higher VM stresses values found in position 1 of the gait are located on the right side and could be due to the asymmetric force distribution as shown in Fig. 2 and Table 2 with more forces applied to the right side of the model. The right superior ramus show its major contribution compared to the right inferior ramus, as confirmed by the principal stress vectors distribution. Bending of the rami by external compression and internal tension comes with internal compression at the greater sciatic notch and sacrum. Considering position 2, both diagonally opposed right superior and left inferior rami significantly contribute to load transfer thanks to the pubic symphysis. In both positions for the intact pelvis, forces are distributed between the anterior and posterior structures of the pelvic ring thereby connecting the spine and the lower extremities. Fracture of the rami was chosen at the superior location, because of findings indicating more injuries at the superior ramus than the inferior (Hill et al. 2001) and due to higher stresses found in this location within the intact numerical model (case A in Fig. 5). The presence of the superior ramus fracture (case B in Fig. 5) alters anteriorly and posteriorly proper distribution in both positions: loads are significantly directed towards the inferior ramus and also internally on the posterior structures as illustrated by VM stresses for both position 1 and 2. With this superior ramus fracture, more VM stresses are applied to the right inferior ramus on position 1 than in position 2. Loads on the posterior structures at the region of the SI joints are moved from right to left side because of the shift of physiological loads between right and left during gait. With same-sided superior and inferior rami fractures (case C in Fig. 5), forces do not cross anymore the front of the pelvis because of the total rupture and are hence directed backwards. The significant increase of stresses on the back structures is linked to this phenomenon and is commonly seen on medical imaging with growing of a sacral compression fracture on one side.

Repeating the study with additional geometries of pelvis could strengthened the observations made in FE analysis on the primordial role of pubic rami on pelvic load distribution. Nevertheless, no study in literature was found to consider different pelvises given the time-consuming and skill-requiring tasks that are segmentation and FE modelling / analysing. In the present study, it is believed that the two steps approach combining inverse dynamics and FE is already a significant step forward to account for physiological load distribution within the pelvis.

Figure 5 summarizes the important stress values shown in Fig. 3 at the three most interesting locations of superior and inferior rami and sacrum for position 1 and 2 for the three analysed models of intact pelvis, superior ramus fractured and both superior and inferior rami fractured. In Ott et al. (2010) and Ott (2007), the ultimate tensile strength Rm of cortical bone can be found and values between 80 MPa and 120 MPa are documented. A high scatter for this strength Rm was detected, as well as for the elongation of break Aϵ[1.5%; 4%]. Highest values for strength and ductility were measured for subjects between 20 and 30 years. The endurance limit, i.e. the maximum stress amplitude a material can withstand for an infinite number of loading cycles is approximately for metals 40% of Rm but also depending additionally on mean stress and type of loading, e.g. bending, torsion, tension, etc. This order of magnitude is presented by the hatched area in Fig. 5, only to indicate a range (from 32 MPa to 48 MPa, respectively 40% from 80 MPa and 120 MPa) for this relevant strength. The gait induces cyclic loadings in the pelvis and Fig. 5 highlights that subsequent fatigue fractures are probable, once a first failure took place.

In Fig. 3 case C, a frontal opening of the anterior pelvic ring can be observed because of the forces exerted by the muscles. Clinically, this is not a realistic scenario but it illustrates the instability of the entire pelvis. It is believed that for patients with fractures the gait would be slightly adapted to reduce pain eventually providing changes in muscular activities. In the present study, it was decided to consider the worst-case scenario and to apply the same forces for comparison purposes with the fractures being the only differences between models.

## Conclusion

The study investigates the loads transmission within the pelvic ring under physiological loadings of the gait. Active muscle forces and joints reaction forces were applied to a FE model to approach the real charging. Because of anterior pelvic ring playing an important role in stability of the pelvis, a superior ramus fracture altered anteriorly and posteriorly the load distribution. The complete anterior rupture transferred the loads directly to the back, creating high stresses and potentially a compression fracture at the sacrum. Close links between numerical and clinical observations in non-fractured and fractured configurations strengthen our study and the use of numerical tools in orthopaedic research for investigation in such problems.
